# Optical properties of Weyl semimetals

**DOI:** 10.1093/nsr/nwy164

**Published:** 2018-12-28

**Authors:** Joel E Moore

**Affiliations:** 1 Department of Physics, University of California, Berkeley, USA; 2 Materials Science Division, Lawrence Berkeley National Laboratory, USA

The behavior of electrons in ordinary metals is dominated by physics near the Fermi surface. Indeed, many of the first successes of the quantum theory of solids, such as understanding the large specific heat of metals, followed from applications of the Fermi surface concept. Great effort has been devoted in recent years to semimetals in which the Fermi surface shrinks to a set of Dirac or Weyl points, where four or two bands cross respectively. 3D crystals with both kinds of band structure have been found in recent years, which completes a search that began in the 1930s with the work of Conyers Herring.

The focus of this article is on the optical properties of Weyl semimetals including TaAs and related compounds, for which much of the initial theoretical [1] and experimental [2] work was carried out in China; given space limitations, we refer the reader to longer reviews [3] for a fuller treatment of the basics and literature. The unusual electronic structure of Weyl semimetals leads to novel optical properties that may be the clearest demonstration of how these materials differ from ordinary metals and insulators. Experiments have already shown strong second-harmonic generation and photocurrent effects in the most studied Weyl material TaAs, and these may lead to optical applications of this and related materials. Theoretical work on topological materials is contributing to improved understanding of non-linear optics in more ordinary solids.

The notion of a Weyl fermion was introduced soon after Dirac’s famous theory of the electron. Hermann Weyl pointed out that, in a relativistic theory, massless fermions can have a fixed helicity, while massive fermions cannot, because boosting to a frame moving faster than the fermion will reverse its helicity. In solid-state terms, this means that a system with broken inversion or time-reversal symmetry can support two-band crossings. Corresponding to the fixed helicity is a topological invariant, the integer flux of Berry curvature through a small sphere surrounding the Weyl point [4]. Weyl points hence have an integer topological ‘charge’, and the total charge of all Weyl points in a crystal is zero (the Nielsen–Ninomiya theorem). Their topological charge leads to a Fermi arc surface state [5] whose observation in experiment is often the first signature of a new Weyl material.

A considerable challenge has been to identify unique properties beyond band structure that result from the existence of Weyl points. Such properties would be analogous to the quantized Hall effect in 2D materials or to the quantized magnetoelectric polarizability in 3D topological insulators, which define those states beyond one-particle physics. One motivation for this perspective is to explain why non-linear optics is a particularly valuable probe. Linear response in a single electromagnetic field is believed not to lead to unique behavior in Weyl materials. This is perhaps not too surprising since the quantum Hall effect involves both **E** and **B** fields, as does the quantized magnetoelectric effect. One linear response that was conjectured for Weyl materials, the ‘chiral magnetic effect’ (distinct from the chiral anomaly, which is an **E** · **B** effect), turns out to be related to natural optical activity or gyrotropy, and to be controlled by the orbital magnetic moment of Bloch electrons rather than by band topology [6,7]. Non-linear responses, however, including standard non-linear optical properties, are already verified to be exceptionally strong in Weyl semimetals [8–10], in addition to having unique features of topological origin.

It may be useful to recall some relevant basic facts about the optical properties of solids. How a metal responds to electromagnetic fields can be intuitively divided into ‘intraband’ and ‘interband’ effects. Light of low frequency and long wavelength can be treated by the semiclassical equations of motion in a normal metal, once these equations are extended to include Berry curvature and orbital moment effects. A true semimetal with point Fermi surfaces is a special case where semiclassical behavior need not apply, since the Fermi surface changes dramatically under even a small change in Fermi energy. (This is a qualitative difference between Weyl and Dirac semimetals and common semimetals such as bismuth, which have small electron and hole Fermi surfaces.) Hence considerable effort has gone into quantum calculations of the optical response of Weyl semimetals [11–13], which in general depend sensitively on wave function properties such as Berry curvature.

The possible optical responses can be narrowed down simply on symmetry grounds, however. The most studied Weyl semimetals break inversion symmetry but do not break time-reversal symmetry. In such materials, an incident source of light at frequency ω can generate a second-harmonic generation (SHG) signal at 2ω and a DC (zero-frequency) photocurrent. Additional information about the crystalline symmetry is necessary to determine the allowed components of these second-order optical responses. The TaAs family of materials is not just inversion-breaking but polar, i.e. the crystalline symmetries allow a non-zero vector, which in an insulator could be viewed as the ground-state electrical polarization. GaAs is an example of a material that breaks inversion and has strong SHG but does not permit a vector because of its rotational symmetries. Interest in the non-linear optics of Weyls was triggered in part by the experimental observation [8] that TaAs has SHG approximately ten times larger than GaAs at fundamental λ ≈ 800 nm, dominated by the component $\chi ^{(2)}_{zzz}$ for which applied field and generated current are both along the polar axis. At longer wavelengths the response is even stronger [14].

Photocurrent is another second-order optical effect and is directly relevant to photovoltaic applications. Strong photocurrent effects have been observed in TaAs [9,10], allowed microscopically by tilting of the Weyl cones [11], and also in type-II Weyl semimetals [15]. It is not yet resolved whether the large second-order responses in TaAs originate directly from its Weyl points or from other properties such as strong asymmetry (‘skewness’) in its Wannier functions [14], which controls the total second-order response in some cases via a sum rule. Weyl points are predicted to have at least one very special optical property, but this is canceled out from symmetry in some materials. For example, although it is non-centrosymmetric, TaAs has a number of other crystalline symmetries that highly constrain the allowed components of its second-order response (and allow separation of thermal effects [10]): its mirror symmetries mean that every energy with positively charged Weyl points has negatively charged Weyl points as well.

Energy-split Weyl semimetals, and also some 3D Rashba compounds, have positive and negative Weyl points at different energies (Fig. 1), and the low crystalline symmetry in these materials allows the circular photogalvanic effect (CPGE), a photocurrent whose sign depends on the sense of circular polarization of the incident light [16]. In an energy-split Weyl semimetal, the rate of CPGE current injection is predicted to be quantized for some frequencies and independent of microscopic parameters, since the strength of current injection divided by the light intensity is a combination of material-independent constants:

(1) \begin{equation*} {1 \over 2 I} \left({dj_{\circlearrowright }\over dt} - {dj_{\circlearrowleft } \over dt} \right) = {2 \pi e^3 \over h^2 c \epsilon _0} C_n. \end{equation*}

Here *C*_*n*_ is a topological integer describing the net Weyl charge below the Fermi surface. Experimental searches for this CPGE are underway: a time-domain experiment could observe the quantized injection rate directly, while the steady-state CPGE photocurrent will additionally depend on the current relaxation rate in the material. Two other active directions involve magnetic fields or materials. One is the search for optical effects in magnetic Weyl semimetals that break time-reversal symmetry either instead of or in addition to inversion symmetry. Another is the experimental investigation of magneto-optical effects that might give clearer evidence for the chiral anomaly of Weyl fermions, as predicted theoretically [17,18]. It seems clear already that non-linear optics is an unexpectedly interesting feature and probe of this exciting family of materials.

**Figure 1.**
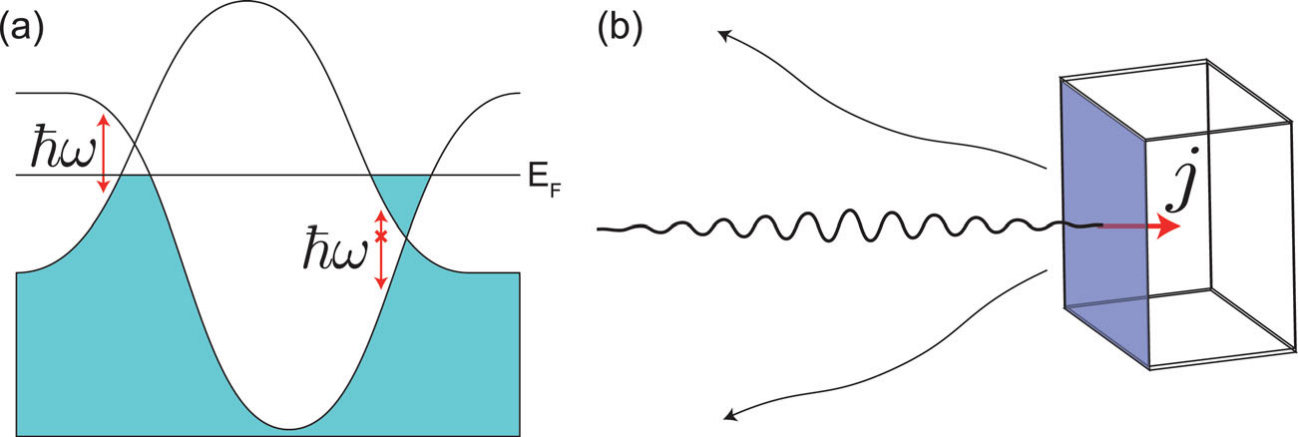
Schematic illustrations of how the circular photogalvanic effect (CPGE) for a single Weyl node quantified in Eq. 1 can be measured in energy-split Weyl semimetals. (a) For a hypothetical material with two Weyl nodes of opposite charge at different energies, there is a range of incident photon frequencies for which optical transitions are allowed across one node but blocked by Pauli exclusion at the other node. The result is that the CPGE is dominated by one node (in this figure, the left node) and approaches its quantized value. (b) A time-domain experiment allows measurement of the low-frequency pulse (outward arrows) produced by the current injected normal to the sample surface by an incident pulse of circularly polarized light. Illustration concept from A. Grushin (Grenoble).

## References

[1] Weng H , Fang C, Fang Z et al. Phys Rev X 2015; 5: 011029.

[2] Lv BQ , Weng HM, Fu BB et al. Phys Rev X 2015; 5: 031013.

[3] Vafek O , Vishwanath A. Ann Rev Condens Matter Phys 2014; 5: 83–112.

[4] Murakami S . New J Phys 2007; 9: 356.

[5] Wan X , Turner AM, Vishwanath A et al. Phys Rev B 2011; 83: 205101.

[6] Ma J , Pesin DA. Phys Rev B 2015; 92: 235205.

[7] Zhong S , Moore JE, Souza I. Phys Rev Lett 2016; 116: 077201. 26943554 10.1103/PhysRevLett.116.077201

[8] Wu L , Patankar S, Morimoto T et al. Nat Phys 2017; 13: 350–5.

[9] Ma Q , Xu S-Y, Chan C-K et al. Nat Phys 2017; 13: 842–7.

[10] Osterhoudt GB , Diebel LK, Yang X et al. arXiv:1712.04951.

[11] Chan C-K , Lindner NH, Refael G et al. Phys Rev B 2017; 95: 041104.

[12] Chan C-K , Lee PA, Burch KS et al. Phys Rev Lett 2016; 116: 026805. 26824561 10.1103/PhysRevLett.116.026805

[13] de Juan F , Grushin AG, Morimoto T et al. Nat Commun 2017; 8: 15995. 28681840 10.1038/ncomms15995 PMC5504287

[14] Patankar S , Wu L, Lu B et al. Phys Rev B 2018; 98: 165113.

[15] Ma J , Gu Q, Liu Y et al. arXiv:1806.08508.

[16] Moore JE , Orenstein J. Phys Rev Lett 2010; 105: 026805. 20867727 10.1103/PhysRevLett.105.026805

[17] Song Z , Zhao J, Fang Z et al. Phys Rev B 2016; 94: 214306.

[18] Kharzeev DE , Kikuchi Y, Meyer R et al. Phys Rev B 2018; 98: 014305.

